# Protein folding and conformational stress in microbial cells producing recombinant proteins: a host comparative overview

**DOI:** 10.1186/1475-2859-7-11

**Published:** 2008-04-04

**Authors:** Brigitte Gasser, Markku Saloheimo, Ursula Rinas, Martin Dragosits, Escarlata Rodríguez-Carmona, Kristin Baumann, Maria Giuliani, Ermenegilda Parrilli, Paola Branduardi, Christine Lang, Danilo Porro, Pau Ferrer, Maria Luisa Tutino, Diethard Mattanovich, Antonio Villaverde

**Affiliations:** 1 University of Natural Resources and Applied Life Sciences Vienna, Department of Biotechnology, Vienna, Austria; 2 VTT Technical Research Centre, Espoo, Finland; 3 Helmholtz Center for Infection Research, Braunschweig, Germany; 4 Autonomous University of Barcelona, Institute for Biotechnology and Biomedicine, Department of Genetics and Microbiology, and CIBER-BBN Network in Bioengineering, Biomaterials and Nanomedicine, Barcelona, Spain; 5 Autonomous University of Barcelona, Department of Chemical Engineering, Barcelona, Spain; 6 University of Naples Federico II, School of Biotechnological Sciences, Naples, Italy; 7 University of Milano-Bicocca, Department of Biotechnology and Bioscience, Milan, Italy; 8 Technical University Berlin, Faculty III, Institute for Microbiology and Genetics, Berlin, Germany

## Abstract

Different species of microorganisms including yeasts, filamentous fungi and bacteria have been used in the past 25 years for the controlled production of foreign proteins of scientific, pharmacological or industrial interest. A major obstacle for protein production processes and a limit to overall success has been the abundance of misfolded polypeptides, which fail to reach their native conformation. The presence of misfolded or folding-reluctant protein species causes considerable stress in host cells. The characterization of such adverse conditions and the elicited cell responses have permitted to better understand the physiology and molecular biology of conformational stress. Therefore, microbial cell factories for recombinant protein production are depicted here as a source of knowledge that has considerably helped to picture the extremely rich landscape of in vivo protein folding, and the main cellular players of this complex process are described for the most important cell factories used for biotechnological purposes.

## Review

One of the main bottlenecks in recombinant protein production is the inability of the foreign polypeptides to reach their native conformation in heterologous host cells, which usually results into their prevalence in the insoluble cell fraction. The unusually high and non-physiological rates of recombinant protein production and the occurrence of significant amounts of misfolded protein species drive the cells to a global conformational stress condition. This situation is characterized by a series of individual physiological responses provoked in order to minimize any toxicity of misfolded protein species and to restore cellular folding homeostasis. The generalized use of microbial cell factories for biological synthesis of proteins and the growing interest in the physiological aspects of conformational stress have converted recombinant cells into schools of protein folding, from which scientists are learning about the cell-protein relationships during the complex process of in vivo protein folding.

The purpose of this review is to summarize the major concepts of the cell biology of protein folding. For that, eukaryotic cells, illustrated by yeasts and filamentous fungi are dissected regarding the mechanics and composition of their folding machinery, misfolding stress responses and strategies to cope with conformational stress. The complexity of the folding, trafficking and secretion machineries of these cell factories is presented versus the relatively simple folding scheme in bacterial cells such as *Escherichia coli *that are also common hosts for recombinant protein production. Despite the existing obvious differences, evolutionary conserved physiological traits regarding folding stress can be identified when comparing eukaryotic and prokaryotic hosts. Furthermore, practical implications of all these findings to improve protein production processes are discussed in their biotechnological context.

## Protein folding and conformational stress in eukaryotic cells

**Figure 1:**
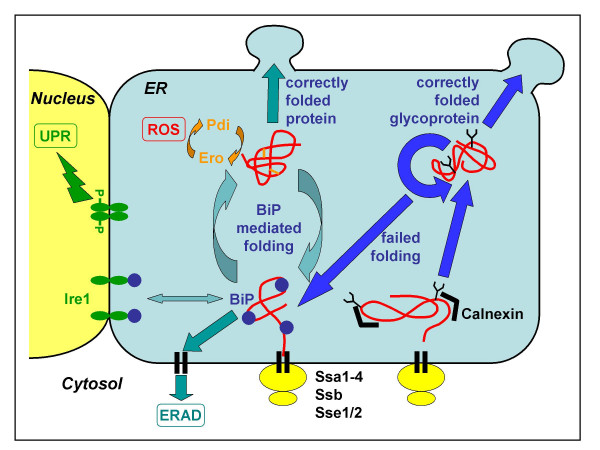
**Schematic representation of protein folding, quality control, degradation and secretion in yeast (as an example for lower eukaryotic cells)**. Secretory proteins are transported into the ER through the Sec61 translocon complex of the ER membrane either co-translationally or post-translationally. In the latter case, cytosolic chaperones (Ssa1-4, Ssb, Sse1/2) support solubility and prevent aggregation of the polypeptide chains. After translocation to the ER, nascent polypeptides are bound by BiP and mediated to mature folding in an ATP-dependent cyclic process of release of and binding to BiP. The formation of correct disulfide bonds is mediated in a cycle of Pdi and Ero activity, which may lead to the formation of reactive oxygen species (ROS). Correctly folded protein is released to transport vesicles, while prolonged BiP binding, indicating misfolding, leads to retrograde translocation to the cytosol and proteasomal degradation (ERAD). Nascent glycoproteins are bound by calnexin and mediated to correct folding and processing of the N-glycans. Failed folding leads to binding by the BiP complex and targeting to ERAD, while correctly folded and processed glycoproteins are released to transport vesicles. Prolonged binding of BiP to partially misfolded proteins leads to the induction of the unfolded protein response (UPR), mediated by Ire1 (see also figure 2).

**Figure 2:**
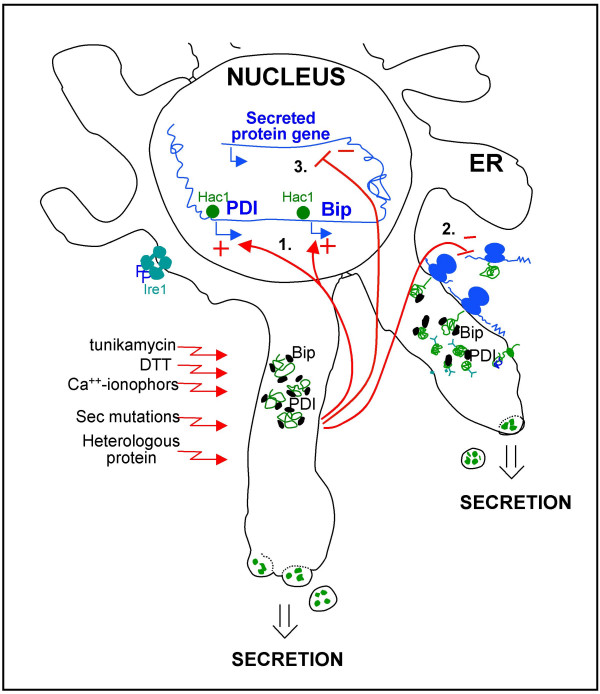
**Schematic representation of secretion stress responses in eukaryotes **Secretory proteins are translocated to the ER either during their translation or post-translationally. Folding of these proteins in the ER can be disturbed by environmental factors or it can be inhibited experimentally by agents inhibiting protein folding like dithiothreitol (DTT) and Ca-ionophores or agents inhibiting glycosylation like tunicamycin. It has been observed that foreign proteins often do not fold well and cause conformational stress. Several responses of the cell to impaired protein folding in the ER have been discovered: 1.) Unfolded protein response (UPR). Genes encoding folding helpers like the chaperone Bip and the foldase protein disulfide isomerase Pdi, and a large number of other genes involved in other functions of the secretory pathway are induced. The proteins Ire1 and Hac1 involved in this signal transduction pathway are shown in the figure. 2.) Translation attenuation. The translation initiation factor eIF2 alpha is phosphorylated, and subsequently translation initiation is inhibited. This reduces the influx of proteins into the ER. This response is only known from mammalian cells. 3.) Repression under secretion stress (RESS). The mRNA levels of genes encoding secreted proteins are down-regulated during ER stress. This response has been discovered in filamentous fungi, but there is evidence for its occurrence in plants.

Yeasts and filamentous fungi are among the most frequently used eukaryotic cell systems for recombinant protein production, in part due to the performance of post-translational modifications that bacteria cannot perform, that are, in most cases, required for proper protein activity. In eukaryotic cells, endoplasmatic reticulum (ER) resident proteins are responsible for correct protein folding. The list of such folding-assistant proteins includes calnexin, chaperones of the hsp70 and hsp90 families (e.g. BiP/Grp78, Grp94), the protein disulfide isomerases (Pdi) which catalyze the formation of disulfide bonds and the peptidyl-prolyl-isomerases. Some of the post-translational modifications such as N-glycosylation are initiated in the ER lumen. Both natural and recombinant proteins are only exported to the Golgi by vesicular transport when their correct conformation has been assured by a glucose-dependent surveillance mechanism of the ER. Unless there is a differing signal, proteins intended for secretion are directed from the Golgi to the outside of the plasma membrane by specific transport vesicles [1,2]. A schematic overview of the protein folding processes is presented in Figure 1, while the responses to secretion stress are summarized in Figure 2.

The protein folding process and subsequent secretion is a rather complex process involving many interacting participants. Due to this interdependence, genetically increasing the rate of one step can lead to rate-limitation of another one, which can then become the bottleneck of the expression system. Moreover, in most cases the rate limiting step in the eukaryotic secretion pathway has been identified to be the exit of proteins from the ER [3]. Linked to this control point is a mechanism called ER-associated protein degradation (ERAD), which is responsible for the retention of misfolded or unmodified non-functional proteins in the ER and their subsequent removal. Protein degradation is executed by linking the misfolded protein to ubiquitin after it has been re-translocated into the cytosol through the same ER translocon pore where it had been imported. The ubiquitin-marked protein is then recognized and degraded by the 26S proteasome in the cytosol (recently reviewed by [4,5].

Two quality control systems in the ER ensure that only correctly folded, modified and assembled proteins travel further along the secretory pathway. The UDP-glucose:glycoprotein glucosyltransferase (UGT) is a central player of glycoprotein quality control in the ER (reviewed among others by [6]). After addition of the core glycan (GlcNac2-Man9-Glc3) to specific asparagine residues of the nascent polypeptide, the three terminal glucose residues have to be clipped off before the protein can exit the ER. Non-native polypeptides are tagged for reassociation with the ER-lectin calnexin by readdition of the terminal glucose onto the N-glycan mediated by UGT. This enzyme specifically recognizes and binds to molten globule-like folding intermediates, thereby acting as sensor of the protein folding status. Re-glucosylation of erroneous glycoproteins prevents their release from the calnexin cycle and subsequent secretion. Upon persistent misfolding, N-glycosylated polypeptides are slowly released from calnexin and enter a second level of retention-based ER quality control by aggregating with the BiP chaperone complex [7]. This correlates with the loss in the ability to emend misfolding. The BiP complex is involved in co-translational translocation of the nascent polypeptide into the ER lumen and preferentially binds to hydrophobic patches. Prolonged binding to either calnexin or the BiP complex targets the polypeptides to the ERAD, however, the exact mechanisms remain elusive (reviewed by [6]). The fact that accumulation of proteins in the ER is able to influence the synthesis of foldases and chaperones such as BiP and Pdi by transcriptional activation in the nucleus lead to the conclusion early on that there must be an intracellular signalling pathway from the ER to the nucleus, called the unfolded protein response (UPR) (for reviews see [8,9]).

After having passed ER quality control successfully, proteins intended for secretion have to be transported to the Golgi network. Specialized cargo vesicles that selectively incorporate these proteins bud from the ER and are targeted to the Golgi membrane by the activity of the coat protein complex II (COPII). In the Golgi network proteins undergo additional post-translational modifications and are subjected to sorting mechanisms that finally target them to their final destination. Possible trafficking routes include direction to the plasma membrane, to the endosomal compartments, to the vacuole, as well as retrograde transport to the ER (review by [10]). Secretory proteins are then delivered to the cell surface by specialized post-Golgi secretory vesicles that dock to and fuse with the plasma membrane. The process called exocytosis includes targeting of the secretory vesicles to the appropriate membrane mediated by the Exocyst, a multiprotein complex, and by interaction of the v-SNAREs (vesicle, in yeast: Snc1/2 proteins) and t-SNAREs (target membrane; Sso1/2p and Sec9p) and release of the cargo proteins outside the cell after fusion of the secretory vesicle with the plasma membrane.

### Impact of the environment on folding and folding stress

During the recent years, it has become evident that a variety of metabolic and environmental stresses may have a strong impact on recombinant protein production. Both types of stress factors occurring during industrial production processes in yeasts, along with potential metabolic and cell engineering approaches to overcome production constraints, were reviewed in Mattanovich et al. [11]. Among environmental factors that affect protein folding and secretion, especially temperature, low pH, high osmolarity and oxidative stress may play an important role.

While many studies have been performed on optimizing fermentation conditions for maximum specific productivity in yeasts, data correlating increased product yields to improved protein folding and secretion mechanisms are still missing. Similar reports regarding the impact of cultivation conditions on protein production in filamentous fungi remain scarce and usually limited to case studies [12-14]. Wang et al. [15] reviewed the impacts of bioprocess strategies on recombinant protein production in filamentous fungi, and concluded that the major effect of the environmental changes correlates to varying morphological forms, which exhibit different secretory capacities.

#### Temperature

Temperature has a profound impact on cell metabolism and abundance/regulation of folding-related genes/proteins (hsp70 family, ER-membrane proteins, etc.). Lowering the cultivation temperature from 30 to 20–25°C has been reported to increase product titers in yeasts in several cases [16-19]. While it may be speculated that a lower growth temperature is leading to lower specific growth rates, thus enabling folding of the recombinant proteins at a lower rate, it was shown recently in chemostat cultures that actually gene regulatory events take place. In continuous cultures of *Pichia pastoris *expressing a human antibody Fab fragment specific productivity of the heterologous protein was significantly increased during the chemostat process at lower temperature (1.4-fold on average). Several genes related to protein targeting to the ER and folding (*SSA4*, *SEC53*, *KAR2*, *ERO1*) and core metabolism genes were found among the genes down-regulated at 20°C, as were the product genes [20]. Transcription of genes involved in the regulation of vesicular transport, exocytosis, ER-associated protein degradation as well as markers for response to oxidative and hyperosmotic stress was enhanced in comparison to 25°C steady state. The reduction in transcriptional activity of the core metabolism is a likely explanation for the reduced mRNA levels of the product genes (LC and HC), which were under control of the glycolytic GAP (glyceraldehyde 3-phosphate dehydrogenase) promoter. The authors hypothesized that at lower temperature a reduced amount of folding stress is imposed on the cells, consequently leading to a higher rate of correctly folded product. Although lower temperature has been shown to improve protein secretion rates, this still depends on the nature of the heterologous protein. Production of a hyperthermophilic enzyme was improved by cultivation at higher temperature (40°C) in *Saccharomyces cerevisiae*, thereby reducing ER folding stress [21].

Additionally to regulatory events, many positive effects of temperature shifts on protein production might be linked to cell wall composition (porosity) and cell cycle. Indeed, increased levels of chitin and cell wall linking beta-glucans have been determined in yeast cells grown at 37°C compared to 22°C in batch cultures [22].

Generally, it turns out that cultivation at an optimized temperature is one of the crucial parameters for improved specific productivity, as it is likely to direct carbon fluxes towards heterologous protein production, and maintains the cells in the more secretion competent phases of the cell cycle.

#### Oxygenation

Redox processes play a major role in heterologous protein production, both related to the oxidation of the product to form disulfide bonds, and to oxidative stress of the host cell during cultivation. Cultivation of methylotrophic yeasts like *P. pastoris *on methanol leads to significant oxidative stress, which may be relieved by the addition of antioxidants like ascorbic acid [23]. Similarly, the expression of antioxidant enzymes like superoxide dismutase was reported to relieve oxidative stress [24].

Apart from environmental stressors, oxidative stress can be imposed on the host cells by intrinsic factors such as leakage in the respiratory pathway, beta-oxidation of lipids, or accumulation of misfolded protein in the ER. There is strong evidence that oxidative stress is connected to growth temperature. While in most cases lower growth temperature results in lower oxidative stress, Gasser et al. [20] showed that the genes coding for the key regulatory enzymes of both the cell redox homeostasis (thioredoxin reductase *TRR1*, thioredoxin peroxidase *TSA1*, glutathione oxidoreductase *GLR1*) and osmoregulation (mitogen-activated protein (MAP) kinase *HOG1*) were induced at the lower temperature where higher secretion rates occur. Generally, the secretory pathway compartments maintain a higher oxidized status compared to the cytosol in order to enable disulfide-bond formation. Finally the electrons generated during the oxidative folding cycles are transferred to molecular oxygen and may lead to the formation of reactive oxygen species [25].

Interestingly, it was shown recently that very low oxygen supply enhances the secretion rate of heterologous proteins in *P. pastoris *significantly, which led to the development of a hypoxic fed batch strategy with over 2-fold increased productivity [26].

#### Osmolarity

So far no clear connection between medium osmolarity and protein folding has been established. Previous data indicate that the response is extremely transient [27]; and even less is known of the effect of osmolarity on heterologous protein production. Mager and Siderius [28] describe temporary cell growth arrest (either at G1 or G2/M) upon hyperosmotic stress conditions accompanied by the induction of the high osmolarity glycerol (HOG) kinase pathway in *S. cerevisiae*. Intracellular glycerol levels are increased in order to adjust osmo-balance through the modification of cell wall integrity. Unlike in animal cells where an osmotic shock leads to increased exocytosis [29], and hyperosmotic GS-NS0 mammalian cells that exhibit an increased specific production rate (albeit decreased growth rate) as compared with iso-osmotic cultures [30], osmo-regulated secretion behaviour in fungi remains unproven. In methanol grown *P. pastoris *cells, salt stress prior to induction was shown to have a positive effect on single chain antibody scFv titers [19], while Lin et al. [18] reported a negative effect of salt supplementation on the secretion of an Fc fusion protein.

#### pH

Osmolarity and pH seem to trigger highly interrelated responses. From an industrial point of view the main desired effect of low pH is to reduce the activity of host proteases which can lead to severe protein degradation (reviewed among others by [31]), but no uniform picture has been assigned to the correlation of pH and protease activity in the culture broth. Both in yeasts and filamentous fungi changing the pH of the culture medium can significantly improve protein yields, however, this effect is most probably not directly associated with improved protein folding mechanisms. On the other hand, lower extracellular pH requires higher energy to maintain intracellular pH values constant/physiological, thereby delaying cell growth and enforcing the cell wall barrier [22,32,33]. Subsequently this more rigid cell wall may diminish secretion efficiency of the pH stressed cells. Lin at al. [18] tested different pH values (ranging from 3.0 to 7.2) during fed batch production of a Fc fusion protein in *P. pastoris *and reported detection of the heterologous protein only at the highest pH of 7.2, however, the authors conclude that the pH optimum is strongly protein and strain dependent.

### Folding stress and heterologous protein production

The ER-resident chaperone BiP (binding protein, in yeast encoded by *KAR2*) belongs to Hsp70 family of heat shock proteins and it is present in the lumen of the endoplasmatic reticulum of all eukaryotes. The yeast homologue is sometimes referred to as Grp78. Binding to BiP prevents the nascent part of secretory or transmembrane proteins from misfolding, until synthesis of the protein is finished. It has been suggested that BiP is not only involved in the translocation of the nascent polypeptides across the ER membrane into the ER lumen, but that it is a key element of an ER-resident quality control mechanism that prevents unfolded proteins from leaving the ER [34]. Other functions associated to BiP are the solubilisation of folding precursors, stabilization of unassembled protein subunits and redirecting misfolded polypeptide chains to the cytosol for ubiquitin-labeling and subsequent degradation by the proteasome (ERAD, ER-associated protein degradation, [35]). Besides a basal constitutive expression level, BiP transcription is induced by the presence of mutant and misfolded proteins in the ER lumen and by stress effects that result in the accumulation of unfolded proteins [36], presumably including the high level expression of heterologous proteins. A saturation of the secretory pathway seems possible, as extractable levels of free folding assistants BiP and Pdi1 decrease when heterologous proteins are overexpressed in *S. cerevisiae *[37]. Kauffman et al. [38] observed an induction of BiP during the expression of a scFv fragment in this yeast species, and Hohenblum et al. [39] have reported increased levels of BiP upon expression of recombinant human trypsinogen in *P. pastoris*. Likewise, biPA and pdiA transcript levels were increased due to heterologous protein overexpression, as well as upon high level secretion of homologous enzymes in filamentous fungi [40-42].

ER-associated protein degradation is a complex process in which misfolded proteins in the ER are redirected to the translocon for retranslocation to the cytosol, where they are subjected to proteasomal degradation. Additionally, excess subunits of multimeric proteins that are unable to assemble are degraded through the ERAD mechanism. According to Plemper et al. [43], the malfolded proteins are retro-translocated through the Sec61-complex translocon pore, through which they had entered the lumen of the ER before, accompanied by ubiquitination at the cytosolic side of the ER membrane. The labeling of substrates destined for degradation by the cytosolic 26S proteasome requires an Ub (ubiquitin) activating enzyme, an Ub conjugating enzyme and an Ub ligase besides ubiquitin itself. In *P. pastoris *three essential components of the ERAD pathway have been shown to be up-regulated upon production of an antibody Fab fragment in correlation to higher protein secretion rates: *HRD1*, coding for an Ub protein ligase, that is able to recruit Ub conjugating enzymes (such as the gene product of *UBC1*) next to the Sec61 translocon pore complex [20].

Prolonged ER retention of misfolded proteins entails repetitive rounds of oxidative protein folding attempts by foldases such as Pdi and consequently results in the generation of reactive oxygen species (ROS). Alleviation of the ER stress is accomplished by the upregulation of the UPR and subsequent induction of the ERAD, however, prolonged UPR induction can also contribute to the stress situation by the accumulation of ROS. In this context, both oxidative stress and ERAD occur in addition to UPR induction when hydrophobic cutinase accumulates in the ER of *S. cerevisiae *[44], while hirudin production in *P. pastoris *lead to increased levels of ROS [23]. Recently it has been shown that overstraining or failure of the ERAD components leads to persistent ER stress conditions and subsequent cell death in both yeasts and higher eukaryotic cells [45,46].

The unfolded protein response pathway is activated by a unique mechanism not known in any other signal transduction pathway (for a recent review see [47]). The sensor protein Ire1p [48] resides in the ER membrane and possesses both kinase and endonuclease activities. When unfolded proteins accumulate in the ER, Ire1p undergoes autophosphorylation and oligomerisation, and catalyses the cleavage of the mRNA encoding the UPR transcription factor, called Hac1/hacA in yeasts and filamentous fungi [49,50] or Xbp1 in mammalian cells [51]. In this way Ire1p initiates an unconventional intron splicing event that has been shown in *S. cerevisiae *to be completed by tRNA ligase [52]. Splicing of yeast *HAC1 *mRNA removes a translation block mediated by the intron [53] and enables formation of the activator protein. For mammalian Xbp1 it has been shown that the unspliced mRNA produces an unstable protein that represses the UPR target genes, whereas the spliced mRNA is translated to a potent, stable activator protein [51]. In the filamentous fungi *Trichoderma reesei, Aspergillus nidulans *[50] and *Aspergillus niger *[54], the hac1/hacA mRNA is truncated at the 5' flanking region during UPR induction, in addition to the unconventional intron splicing. This truncation removes upstream open reading frames from the mRNAs, most probably increasing translation initiation at the start codon of the *HAC1*/*HACA *open reading frame. Kincaid and Cooper [46] identified a novel function of Ire1p in the degradation of mRNAs encoding selected secretory proteins thus avoiding potential overload of the ER and the translocon complex *a priori*.

ER-associated stress responses such as UPR and ERAD were reported to be induced upon overexpression of several heterologous proteins, *e.g*., human tissue plasminogen activator (t-PA) in *T. reesei *[55] and *A. niger *[56], and bovine chymosin in *A. nidulans *[57]. Similarily, overexpression of Fab fragments [20] and *Rhizopus oryzae *lipase [58] revealed UPR induction in *P. pastoris*.

In another layer of ER stress regulation, mammalian cells can attenuate translation initiation during unfolded protein accumulation into the ER, in order to reduce the influx of proteins to the ER. This regulation pathway is initiated by the ER membrane kinase PERK that has some similarity with Ire1 [59]. PERK phosphorylates the translation initiation factor eIF2alpha, resulting in drastic reduction in translation. This mechanism is not known in yeasts or filamentous fungi, and PERK orthologues can not be found in the genomes of the lower eukaryotes. Interestingly, the filamentous fungi *T. reesei *[60] and *A. niger *[61] have an alternative mechanism for controlling the protein influx to the ER. In conditions of ER stress the mRNAs encoding secreted proteins are rapidly down-regulated. This mechanism called RESS (repression under secretion stress) was shown to be dependent on the promoters of the genes encoding secreted proteins, and thus it probably functions at the level of transcription [60]. It has been observed that in *Arabidopsis thaliana *a large number of genes encoding secreted proteins are down-regulated when cells are exposed to ER stress [62], implying the possibility that RESS might also exist in plants.

### Overcoming folding stress for improved protein production

Although promising expectations emerged that increased BiP levels would result in increased folding capacity in the ER, and thus improved secretion rates, the findings were rather inconsistent and unpredictable. Some studies emphasize that overproduction of BiP stimulates protein secretion in *S. cerevisiae *(5-fold increase in secretion of human erythropoietin [63], 26-fold increase in bovine prochymosin [64], 2.5-fold increase in the titer of antithrombotic hirudin due to 2.5 times higher biomass yields [65]). While the secretion level of plant thaumatin in *Aspergillus awamori *was increased up to 2.5-fold compared to a wild type strain due to bipA overexpression [66], the secretory behaviour of the same protein was not affected by overexpression of *KAR2 *in *S. cerevisiae *[64]. According to Wittrup and coworkers, a reduction of BiP levels leads to decreased secretion of foreign proteins, however, no effect was observed upon a 5-fold overexpression of BiP on secretion levels of three different recombinant proteins in *S. cerevisiae *[67], and neither for cutinase in *A. awamori *[68]. Other reports even suggest a negative impact of BiP overexpression, as extracellular levels of *A. niger *glucose oxidase (GOX) decreased 10-fold upon BiP overexpression in *Hansenula polymorpha *[69]. As prolonged binding to BiP seems to direct proteins rather to degradation than to the secretory pathway, it becomes more obvious why the overexpression of this chaperone alone does not result in higher levels of secreted foreign proteins, but can negatively influence expression levels, as reported by Kauffman *et al*. [38] and van der Heide *et al*. [69]. Interestingly, *Pyrococcus furiosus *beta-glucosidase secretion in *S. cerevisiae *is diminished with increased BiP levels, but benefited from higher protein disulfide isomerase (Pdi) levels, although the protein did not contain any disulfide bonds [70], pointing at the chaperone activity of Pdi, as discussed below.

Conesa et al. [71] examined the impact of overexpression of two ER quality control factors, BiP and calnexin, on the secretion of glycosylated *Phanerochaete chrysosporium *manganese peroxidase (MnP) in *A. niger*, as the expression levels of these genes were induced upon recombinant protein production. While BiP overproduction diminished manganese peroxidase secretion levels severely, overexpression of calnexin resulted in a four- to fivefold increase in the extracellular MnP levels. Higher levels of calnexin also showed beneficial effects in mammalian and baculo virus expression systems [72,73]. Recently, the co-overexpression of calnexin was shown to stimulate the secretion of three glycoproteins and one unglycosylated product (HSA) in *H. polymorpha *(2–3 fold on average; [74]). On the other hand, secretion of human serum albumin (HSA) remained unaffected by raising calnexin levels in *Schizosaccharomyces pombe *[75], while in *S. cerevisiae *deletion of the calnexin gene *CNE1 *was reported to enhance secretion of both antitrypsin [76] and unstable lysozymes [77,78].

Protein disulfide isomerase (Pdi) is a multifunctional protein resident in the ER lumen that is responsible for the correct formation of disulfide bonds during oxidative folding and the isomerisation of uncorrectly folded disulfides. Apart from this foldase activity, Pdi also acts as a chaperone. An additional *PDI *gene copy in *S. cerevisiae *successfully improved secretion of human growth factor by 10-fold, of *S. pombe *acid phosphatase by 4-fold [63] and of human lysozyme by around 30–60% [79]. Human lysozyme as well as HSA production could also be enhanced by the same strategy in *Kluyveromyces lactis *(1.8 fold and 15 fold, respectively; [80,81]). Both *S. cerevisiae PDI1 *and the *P. pastoris *own homolog were proven to be functional in *P. pastoris *by facilitating secretion of the human parathyroid hormone (hPTH, [82]), human anti HIV1 2F5 Fab [83], and *Necator americanus *secretory protein Na-ASP1 [84], the latter reporting a correlation between the secretory enhancement and the *PDI *copy number. Generally, no clear picture emerged from the co-overexpression of the two folding helpers, BiP and Pdi. Whereas synergistic action of BiP and Pdi was suggested regarding the improvement of the secretion of various single chain fragments (scFv) in *S. cerevisiae *[85], a 2-fold increase in secretion of the A33scFv in *P. pastoris *was only achieved by additional copies of *KAR2*, but not *PDI*, and not by the combination of both [86], in analogy to the antagonistic effect observed in CHO cells [87]. Coexpression of *KAR2*, *PDI1 *or *SSO2 *exhibited no effect on secretion of gamma-Interferon (IFNgamma) in *H. polymorpha *[88]. Moreover, coexpression of *cypB*, which encodes a foldase of the ER secretory pathway [89], did no increase production of tissue plasminogen activator (t-PA) in *A. niger*, although t-PA production elicited an UPR response detectable through elevated transcript levels of *bip, pdi *and *cypB *[90]. Thus, it seems that the effect of coexpression of chaperone and foldase genes strongly depends on the properties of the target protein and, moreover, it seems that fine-tuned overexpression of these genes are required to generate a functional secretory network to improve foreign protein overproduction. For example, in *A. niger*, overexpression of *bip *to a certain threshold was beneficial for plant sweet protein thaumatin production, but above this threshold level thaumatin production decreased [66]. Similarly, defined levels of Pdi were required for optimum thaumatin secretion in *A. niger *[91].

The flavoenzyme Ero1 is required for oxidation of protein dithiols in the ER. It is oxidized by molecular oxygen and acts as a specific oxidant of protein disulfide isomerase (Pdi). Disulfides generated de novo within Ero1 are transferred to Pdi and then to substrate proteins by dithiol-disulfide exchange reactions [92]. Duplication of either *KlPDI1 *or *KlERO1 *genes led to a similar increase in HSA yield in *K. lactis*, while duplication of both genes accelerated the secretion of HSA and improved cell growth rate and yield. Increasing the dosage of *KlERO1 *did not affect the production of human interleukin 1beta, a protein that has no disulfide bridges [93].

Finally, another approach to stimulate the secretory pathway concertedly is to overexpress the unfolded protein response (UPR) activating transcription factor Hac1. Transcriptional analyses in *S. cerevisiae *revealed that up to 330 genes are regulated by Hac1, most of them belonging to the functional groups of secretion or the biogenesis of secretory organelles (e.g. ER-resident chaperones, foldases, components of the translocon). Interestingly, genes encoding proteins involved in protein degradation, vesicular trafficking, lipid biogenesis and vacuolar sorting are also induced by Hac1 [94]. In this context, Higashio and Kohno [95] describe the stimulation of ER-to-Golgi transport through the UPR by inducing COPII vesicle formation. The homologs of *S. cerevisiae HAC1 *in *T. reesei *(hac1) and *A. nidulans *(hacA) have been identified [50] and the effects of UPR induction by constitutive overexpression of these genes have been evaluated. The heterologous overexpression of *T. reesei *hac1 in *S. cerevisiae *yielded a 2.4-fold improvement in *Bacillus *α-amylase secretion, and a slight increase in the yeast endogenous invertase as well as in total protein secretion. *S. cerevisiae HAC1 *overexpression was shown to enhance secretion of the endogenous invertase (2-fold), and recombinant α-amylase (70% increase), but did not effect secretion of *T. reesei *EGI, a protein supposed to accumulate in the ER. Disruption of *HAC1 *in *S. cerevisiae *led to a reduced secretion of the two recombinant proteins (α-amylase -75%, EGI -50%), but not of the endogenous invertase [96]. Similar results could also be seen in *A. awamori*, where overproduction of *A. awamori *hacA ameliorated secretion of *Trametes versicolor *laccase and bovine preprochymosin 7-fold and 2.8 fold, respectively [97], and in *P. pastoris*, where heterologous expression of *S. cerevisiae HAC1 *increased the secretion rate of a Fab antibody fragment [83].

### Novel strategies: genome wide-screening

All these approaches are limited to the existing knowledge base. Novel processes might be identified and targeted to improve secretion (including non-UPR regulated genes) through different approaches. In this regard, high throughput flow cytometry and cell sorting are valuable tools to isolate overproducing clones [98]. One approach is to screen overexpression libraries for improved secretion of heterologous protein, which is anchored to the cell surface via agglutinin (Aga2p) and detected by immunofluorescent staining. Shusta et al. [99] showed that the levels of surface-displayed single chain T-cell receptors correlated strongly with the soluble expression of the respective proteins. A 3-fold higher secreting clone could be isolated out of a library potentially as large as 10^8 ^in a couple of weeks [100]. Screening of a yeast cDNA library in *S. cerevisiae *surface display strains identified cell wall proteins, translational components and the folding assistant Ero1 as beneficial for the secretion of various antibody fragments [101]. However, one potential drawback of this high throughput method is that the display efficiency of the protein of interest can be dominated by its fusion partner Aga2p, as BiP and *PDI *overexpression had no effect on surface display levels of the scFvs although they increased soluble expression levels [85].

Furthermore, genome-wide analytical tools like DNA microarrays are regarded as data mining source for physiological effects, stress regulation and host engineering. Sauer et al. [102] have analysed the differential transcriptome of a *P. pastoris *strain overexpressing human trypsinogen versus a non-expressing strain. 13 out of the 524 significantly regulated genes were selected, and their *S. cerevisiae *homologs were overexpressed in a *P. pastoris *strain producing a human antibody Fab fragment [103]. Five previously characterized secretion helpers (*PDI1*, *ERO1*, *SSO2*, *KAR2*/BiP and *HAC1*), as well as 6 novel, hitherto unidentified, factors, more precisely Bfr2 and Bmh2 involved in protein transport, the chaperones Ssa4 and Sse1, the vacuolar ATPase subunit Cup5 and Kin2, a protein kinase connected to exocytosis, proved their benefits for practical application in lab scale production processes by increasing both specific production rates as well as volumetric productivity of an antibody fragment up to 2.5-fold in fed batch fermentations of *P. pastoris *[103].

## Protein folding and conformational stress in prokaryotic cells

**Figure 3:**
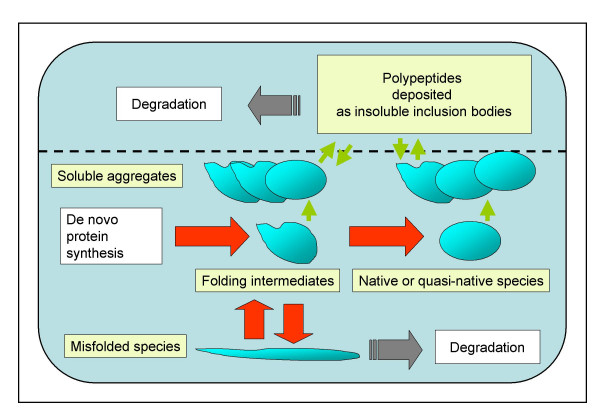
**Schematic representation of protein folding and aggregation in recombinant *E. coli***. After de novo synthesis, a fraction of recombinant proteins (especially heterologous proteins with conformationally complex disulfide bridges) do not reach their native conformation and aggregate as insoluble deposits named inclusion bodies. Protein aggregates already exist in the soluble cell fraction, and can involve native or quasi-native protein species. The main cytoplasmic chaperones involved in the protein folding process (red arrows) include the trigger factor, DnaK, DnaJ, GrpE, GroEL and GroES. Both soluble aggregates and individual protein species can enter the virtual insoluble cell fraction (indicated by a dashed line) and deposit as inclusion bodies, in a fully reversible process (green arrows). Protein release from inclusion bodies is mainly controlled by DnaK, ClpB and IbpA,B. Proteases (lon, ClpP and others) attack both soluble and insoluble species with folding defects. In particular, proteases degrade inclusion body proteins in situ, or through a more complex process intimately related to the protein release process, and therefore, strongly dependent on DnaK.

Since early recombinant DNA times, bacteria (especially *E. coli*) have been the most widely used microorganisms for recombinant protein production due to genetic simplicity, fast growth rate, high cell density production and availability of a spectrum of genetic systems, among others. For production processes being efficient, foreign genes are expressed from plasmids and under the control of inducible promoters, what results into non physiological and unusually high transcription rates. Strong production of recombinant proteins can lead to a stressful situation for the host cell, with the extent of the bacterial stress response being determined by the specific properties of the recombinant protein, and by the rates of transcription and translation [104]. This fact has a clear and profoundly negative impact on productivity and probably protein quality. In addition, recombinant proteins fail, very often, to reach their native conformation when produced in bacteria [105]. This is caused by a coincidence of diverse events impairing protein folding including bottlenecks in transcription and translation, undertitration of chaperones and proteases, improper codon usage and inability of disulfide-bond formation [106,107]. Misfolded protein species usually deposit as amorphous masses of insoluble material called inclusion bodies [108], recorded as by-products of bacterial protein production processes. Inclusion bodies are mainly formed by the deposition of unfolded or partially misfolded protein species that interact through hydrophobic patches unusually exposed to the solvent and with high amino acid sequence homology [109,110]. The specificity in protein aggregation makes inclusion bodies highly pure in composition and therefore enriched in the recombinant protein itself. However, also truncated versions of the recombinant product, other plasmid-encoded proteins, but also defined host cell proteins can get entrapped within bacterial inclusion bodies [111-116]. Moreover, the presence of folding assistant proteins in inclusion bodies [117-119] confirm that specific interactions lead to entrappment of cellular proteins in these aggregates. The high purity of inclusion bodies makes them a convenient source of easily extractable protein that must be refolded in vitro by denaturing procedures, a fact that has been largely exploited for biotechnology purposes [120]. The potential routes of a newly synthesized protein in the bacterial cytosol are illustrated in Figure 3.

Although inclusion bodies are mainly found in the cytoplasm, they occur also in the bacterial periplasm if proteins have been engineered to present a leader peptide for secretion [121]. In fact, a control quality system mostly separated from that acting in the cytoplasm assist folding of secreted proteins in the periplasmic space of gram negative bacteria. This is regulated through the combined activity of two partially overlapping systems, regulated by the alternate σ factor σ^E ^and by the Cpx envelope stress signalling system, that intricately combine the activity of chaperones and proteases [122,123]. However, the simultaneous activation of stress signals in both bacterial compartments upon the production of misfolding prone proteins strongly suggest a close physiological and genetic connection between cytoplasmic and extracytoplasmic systems [124]. The quality control and conformational stress in the periplasmic space has been extensively revised elsewhere [121,125].

Different to the unfolded protein response (UPR) described in eukaryotic cells, the physiological reaction to conformational stress in the bacterial cytoplasm has not received any similar precise name. Transcriptome analysis of recombinant *E. coli *has resulted in a catalogue of genes up-regulated during protein production [126,127]. Among them several heat shock genes have been identified (including those encoding the proteases Lon, ClpP, HslV and HslU, and the chaperones IbpA, IbpB, DnaK, DnaJ, ClpB, HtpG, MopA and MopB among others) but also other ones not directly involved in protein quality (such as YagU, YojH, YbeD and others) and whose precise role remains to be identified. This fact indicates that the conformational stress imposed by protein production is more complex and physiologically distinguishable from that caused by thermal denaturation, namely the heat shock response [128,129], and includes several overlapping stress responses [104] Well characterized stress events have been observed during recombinant protein production such as SOS DNA repair [130] and stringent responses [131], although it is still be solved whether such reactions are directly associated to the prevalence of unfolded or misfolded protein species and the eventual connection with the σ^32^-regulated heat shock response. The expression of some of these stress genes is being used as a convenient marker of conformational stress in recombinant cells [132].

The bacterial conformational stress itself has been poorly characterized from its physiological side. Instead, many efforts have been addressed to a rather practical issue such as minimizing aggregation, what in turn has resulted in a better comprehension of in vivo protein folding processes. Since solubility has been considered for a long time being synonymous with protein quality, increasing the relative yield of soluble protein has been targeted by physicochemical approaches. From already classical studies, it is well known that high temperatures impair protein folding and favour aggregation of the recombinant proteins as inclusion bodies [133,134]. Therefore, reducing the growth temperature has been a general strategy used to minimize inclusion body formation [135-137] that, like other strategies, has rendered moderately positive, but unpredictable and product-dependent results [107]. Fusion of folding-reluctant species to highly soluble homologous or thermostable proteins has in some cases, resulted in moderate enhancement of the passenger protein solubility [106,138,139].

### Chaperones and protein degradation

Folding failures of recombinant proteins produced in *E. coli *is generally attributed to a limitation in the cell concentration of folding assistant elements, which cannot process the newly synthesized aggregation prone polypeptides. This assumption is physiologically supported by the overexpression of chaperone genes, in particular of chaperone genes from the heat-shock protein family, in response to recombinant protein overproduction [126,127,133]. Thus, coproduction of the main heat shock chaperones (specially GroEL and DnaK) together with the target protein has been largely explored as a way to minimize aggregation and to enhance the solubility of the recombinant protein product (reviewed in [140-143]). In many cases, solubility has been significantly enhanced by coexpression of individual chaperone genes, while in others an even negative effect on product stability and host viability has been observed. Selection of the suitable chaperone(s) is still a trial-and-error process. However, more recent results indicate that complete chaperones gene sets rather than individual chaperone genes with synergistic and/or cooperative activities (such as DnaK-DnaJ-GrpE and GroEL-GroES sets) will lead to a more predictable improvement of target protein solubility. [144-147].

Interestingly, when producing enzymes or fluorescent proteins in DnaK^- ^cells, the biological activities and therefore the conformational quality of aggregated polypeptides is much more close to that of soluble versions, compared to wild type cells [148-150]. Furthermore, the overexpression of the *dnaK *gene along with a model GFP recombinant protein dramatically reduces the specific fluorescence of a GFP fusion in both soluble and insoluble versions [151]. This indicates that DnaK directly or indirectly impairs the folding state of the aggregated proteins. In this regard, the production of GFP variants in absence of DnaK results in highly fluorescent inclusion bodies [152]. In these cells, both the protein yield and quality were dramatically enhanced although the solubility is lower than in the wild type, as expected. This occurs by the inhibition of GFP proteolysis mediated by the proteases Lon and ClpP, which participate in the in vivo disintegration of inclusion bodies in absence of protein synthesis [153,154]. Probably, such proteases act coordinately in a disaggregation complex [155-157] in which DnaK, ClpB and IbpAB remove aggregated polypeptides for proteolytic digestion. Therefore, although solubility can be indeed enhanced by high levels of DnaK, GroEL and other chaperones it occurs at expenses of quality and yield, probably by generally stimulating proteolysis [116]. In fact, solubility and conformational quality are not only non coincident parameters [158] but highly divergent protein features [152].

### Disulfide-bond formation in recombinant *E. coli*

Usually, the cytoplasmic space of *E. coli *is a reducing environment. Therefore, disulfide-bonds within proteins are not formed, a fact that represents an additional obstacle for proper folding of many recombinant proteins. There are two approaches to produce disulfide-bonded proteins in *E. coli *expression, namely *in vitro *refolding of inclusion body proteins under appropriate redox conditions [120] or manipulating *in vivo *conditions by either converting the cytoplasm into an oxidizing environment or secreting the protein into the periplasmic space or even further into the culture medium (less reducing environments). Correct disulfide bond formation in the periplasm of *E. coli *is a catalyzed process, where the oxidation of cysteine pairs occurs through the transfer of disulfides from the highly oxidizing DsbA/DsbB proteins to the proof-reading proteins DsbC/DsbD which are able to rearrange non-native disulfides to their native configuration [159]. In particular, overexpression of DsbC has been shown to increase the yield of correctly disulfide-bonded proteins in the periplasm of *E. coli *[160-162]. The co-expression of eukaryotic protein disulfide isomerases in *E. coli *can also favour the formation of disulfide bonds in the periplasmic space [163,164].

Disulfide bond formation in the cytoplasm of *E. coli *can occur when the genes encoding thioredoxin reductase (*trxB*) and glutathione oxido-reductase (*gor*) are inactivated [165,166]. A double-mutant strain containing appropriate mutations, known as Origami, has been used, for example, to generate active variants of tissue-type plasminogen activator [165] and functional antibody fragments in the *E. coli *cytoplasm [167,168]. In some cases, recovery of functional cytoplasmic disulfide-bonded proteins can be further enhanced by coexpressing signal sequence deficient periplasmic chaperones and/or disulfide-bond isomerases such as DsbC [165,167,169,170]. Unfortunately, *trxB gor *mutants exhibit impaired growth characteristics [112,165], but, at least for antibody fragments it has been shown that expression yields of correctly disulfide-bonded proteins in the cytoplasm can be similar to those obtained by secretion into the periplasmic space [171].

### Protein folding and secretion in non-conventional bacterial expression systems

Although *E. coli *is still the most commonly used prokaryotic organism for heterologous protein production, other bacterial hosts are becoming more and more attractive.

Gram-positive Bacilli strains are also frequently employed at industrial level. In contrast to *E. coli*, their outer envelope has no lipopolysaccharides, also called "endotoxins" since they exert a pyrogenic activity in humans or other mammals. Therefore, many pharmaceutically relevant products have been obtained in several strains [172]. In addition, the Bacilli strains are attractive hosts because they have a naturally high secretion capacity, as they export proteins directly into the extracellular medium. Amongst Bacilli species, the protein secretion pathway in *B. subtilis *have been deeply investigated at molecular level and a comprehensive literature survey is reported in [173]. Several bottlenecks in the expression and secretion of heterologous proteins have been highlighted [174]. The secretory pathway of proteins can be divided into three functional stages: the early stage, involving the synthesis of secretory pre-proteins, their interaction with chaperones and binding to the secretory translocase complex; the second stage, consisting in translocation across the cytoplasmic membrane; and the last stage, including removal of the N-terminal signal peptide, protein refolding and passage through the cell wall. A pivotal role in the whole secretion process is played by molecular chaperones [175]. *B. subtilis *has two types of molecular chaperones, intracellular and extra-cytoplasmic molecular chaperones. GroES, GroEL, DnaK, DnaJ and GrpE are intracellular molecular chaperones. Besides being involved in and largely responsible for protein folding and minimizing aggregation, these chaperones maintain pre-proteins (the products to be secreted) in translocation-competent conformations [176]. PrsA is the only known extracytoplasmic folding factor in *B. subtilis*. PrsA is a lipoprotein that consists of a 33 kDa lysine-rich protein part and the N-terminal cysteine with a thiol-linked diacylglycerol anchoring the protein to the outer leaflet of the cytoplasmic membrane [177]. Subsequent folding of a secreted mature protein into a stable and active conformation usually requires PrsA protein. In *prsA *mutants, the secretion and stability of some model proteins is decreased, if not abolished, while overproduction of PrsA enhances the secretion of exoproteins engineered to be expressed at a high level [178].

There is, however, a physiologic limit to the overloading of *B. subtilis *secretory pathway. The massive production of homologous and heterologous exoproteins has been reported to induce a phenomenon called "protein secretion stress response" [179]. The CssRS two-component regulatory system is able to detect the presence of partially folded or unfolded exo-protein intermediates and activates the transcription of several genes, among which a key role is played by *htrAB*. These genes encode two membrane localised serine proteases involved in the proteolysis of aberrant products [180].

Several gene expression systems using non-conventional prokaryotic organisms as host cells have been developed over the last decades. Each bacterial host was generally implemented to overcome defined problems/bottlenecks observed during the recombinant production of specific protein classes in conventional systems, such as *E. coli *and *B. subtilis*. The use of such non-conventional systems is still very limited and largely suffers from the lack of molecular details concerning host physiology and any other phenomenon related to massive recombinant protein production. Notwithstanding, some of them may represent useful model systems to further investigate on the optimization of recombinant protein folding and quality.

In this context, some interest has been raised by the implementation of an Antarctic Gram negative bacterium as production host. *Pseudoalteromonas haloplanktis *TAC125 was isolated from a sea water sample collected in the vicinity of the Dumont d'Urville Antarctic station, in Terre Adélie. It is characterised by fast growth rates, combined with the ability to reach very high cell densities even in uncontrolled laboratory growth conditions and to be easily transformed by intergeneric conjugation [181]. There features made *P. haloplanktis *TAC125 an attracting host for the development of an efficient gene-expression system for the recombinant protein production at low temperatures of thermal-labile and aggregation-prone proteins [182]. Furthermore, it was the first Antarctic Gram-negative bacterium which genome was fully determined and carefully annotated [183].

Since high temperatures have a general negative impact on protein folding due to the strong temperature dependence of hydrophobic interactions that mainly drive the aggregation reaction [184], and favour conformational stress, the production of recombinant proteins at low temperatures represents an exciting model to study the dynamics of protein folding and misfolding and to improve the quality of the products. The growth of *E. coli *below 37°C has been often explored to minimize aggregation but without consistent, protein-irrespective results. Also, the use of suboptimal growth temperatures might negatively affect the biology of the host cell and the performance of the process in undesirable and non predictable ways. Recombinant protein production in psychrophilic bacteria, i.e. at temperature as low as 4°C, may minimize undesired hydrophobic interactions during protein folding, desirably resulting in enhancing the yield of soluble and correctly folded products while operating close to the optimal growth range. Furthermore, with respect to mesophilic cells growing at suboptimal temperatures, psychrophiles contain a full set of folding factors already adapted to assist optimally, when required, protein folding at freezing temperatures.

The efficiency of the cold-adapted expression system was tested by producing several aggregation-prone products in *P. haloplanktis *TAC125, such as a yeast α-glucosidase [182], the mature human nerve growth factor [182], and a cold adapted lipase [185]. All the recombinant products resulted to be fully soluble and biologically competent.

## Concluding remarks

In vivo protein folding is a very complex issue that involves many cellular proteins and physiological responses. During recombinant protein production, conformational stress conditions elicited by the synthesis of aggregation prone polypeptides profoundly alter the physiology of the host cell, triggering mechanisms addressed to manage potentially toxic misfolding protein species and to recover the cell folding homeostasis. The use of different microorganims as factories for recombinant protein production, including yeast, filamentous fungi and bacteria has resulted in dramatic gains of information about the biology of such stress responses, and has provided valuable information to better understand the mechanics of in vivo protein folding and aggregation.

However, so far it has not been possible to create the "perfect folding environment". Especially with respect to industrial protein production processes, the direct impact of altered process conditions on recombinant protein folding remains unclear. Ongoing research in the authors' labs is targeted to elucidate the physiological responses of different eukaryotic and prokaryotic microbial hosts on a genome wide level in order to interrelate environmental stresses to protein folding/aggregation mechanisms and eliminate bottlenecks.

## Competing interests

The author(s) declare that they have no competing interests.

## Authors' contributions

All authors contributed equally to this manuscript, and read and approved the final version.

## References

[B1] Gething MJ, Sambrook J (1992). Protein folding in the cell. Nature.

[B2] Stryer, Lubert (1995). Biochemie. Spektrum der Wissenschaft VerlagsGmbH.

[B3] Shuster JR (1991). Gene expression in yeast: protein secretion. Curr Opin Biotechnol.

[B4] Brodsky JL, Scott CM (2007). Tipping the delicate balance: defining how proteasome maturation affects the degradation of a substrate for autophagy and endoplasmic reticulum associated degradation (ERAD). Autophagy.

[B5] Meusser B, Hirsch C, Jarosch E, Sommer T (2005). ERAD: the long road to destruction. Nat Cell Biol.

[B6] Kleizen B, Braakman I (2004). Protein folding and quality control in the endoplasmic reticulum. Curr Opin Cell Biol.

[B7] Molinari M, Galli C, Vanoni O, Arnold SM, Kaufman RJ (2005). Persistent glycoprotein misfolding activates the glucosidase II/UGT1-driven calnexin cycle to delay aggregation and loss of folding competence. Mol Cell.

[B8] Ma Y, Hendershot LM (2001). The unfolding tale of the unfolded protein response. Cell.

[B9] Patil C, Walter P (2001). Intracellular signaling from the endoplasmic reticulum to the nucleus: the unfolded protein response in yeast and mammals. Curr Opin Cell Biol.

[B10] Nakano A (2004). Yeast Golgi apparatus – dynamics and sorting. Cell Mol Life Sci.

[B11] Mattanovich D, Gasser B, Hohenblum H, Sauer M (2004). Stress in recombinant protein producing yeasts. J Biotechnol.

[B12] Garcia-Kirchner O, Segura-Granados M, Rodriguez-Pascual P (2005). Effect of media composition and growth conditions on production of beta-glucosidase by Aspergillus niger C-6. Appl Biochem Biotechnol.

[B13] Liu CQ, Chen QH, Cheng QJ, Wang JL, He GQ (2007). Effect of cultivating conditions on alpha-galactosidase production by a novel Aspergillus foetidus ZU-G1 strain in solid-state fermentation. J Zhejiang Univ Sci B.

[B14] Wang L, Ridgway D, Gu T, Moo-Young M (2003). Effects of process parameters on heterologous protein production in Aspergilus niger fermentation. Journal of Chemical Technology and Biotechnology.

[B15] Wang L, Ridgway D, Gu T, Moo-Young M (2005). Bioprocessing strategies to improve heterologous protein production in filamentous fungal fermentations. Biotechnol Adv.

[B16] Hackel BJ, Huang D, Bubolz JC, Wang XX, Shusta EV (2006). Production of soluble and active transferrin receptor-targeting single-chain antibody using Saccharomyces cerevisiae. Pharm Res.

[B17] Li Z, Xiong F, Lin Q, d'Anjou M, Daugulis AJ, Yang DS, Hew CL (2001). Low-temperature increases the yield of biologically active herring antifreeze protein in Pichia pastoris. Protein Expr Purif.

[B18] Lin H, Kim T, Xiong F, Yang X (2007). Enhancing the production of Fc fusion protein in fed-batch fermentation of Pichia pastoris by design of experiments. Biotechnol Prog.

[B19] Shi X, Karkut T, Chamankhah M, Alting-Mees M, Hemmingsen SM, Hegedus D (2003). Optimal conditions for the expression of a single-chain antibody (scFv) gene in Pichia pastoris. Protein Expr Purif.

[B20] Gasser B, Maurer M, Rautio J, Sauer M, Bhattacharyya A, Saloheimo M, Penttilä M, Mattanovich D (2007). Monitoring of transcriptional regulation in Pichia pastoris under protein production conditions. BMC Genomics.

[B21] Smith JD, Richardson NE, Robinson AS (2005). Elevated expression temperature in a mesophilic host results in increased secretion of a hyperthermophilic enzyme and decreased cell stress. Biochim Biophys Acta.

[B22] Aguilar-Uscanga B, Francois JM (2003). A study of the yeast cell wall composition and structure in response to growth conditions and mode of cultivation. Lett Appl Microbiol.

[B23] Xiao A, Zhou X, Zhou L, Zhang Y (2006). Improvement of cell viability and hirudin production by ascorbic acid in Pichia pastoris fermentation. Appl Microbiol Biotechnol.

[B24] Li JR, Yu P (2007). Expression of Cu, Zn-superoxide dismutase gene from Saccharomyces cerevisiae in Pichia pastoris and its resistance to oxidative stress. Appl Biochem Biotechnol.

[B25] Tu BP, Weissman JS (2004). Oxidative protein folding in eukaryotes: mechanisms and consequences. J Cell Biol.

[B26] Baumann K, Maurer M, Dragosits M, Cos O, Ferrer P, Mattanovich D (2008). Hypoxic fed batch cultivation of Pichia pastoris increases specific and volumetric productivity of recombinant proteins. Biotechnol Bioeng.

[B27] Gasch AP, Spellman PT, Kao CM, Carmel-Harel O, Eisen MB, Storz G, Botstein D, Brown PO (2000). Genomic expression programs in the response of yeast cells to environmental changes. Mol Biol Cell.

[B28] Mager WH, Siderius M (2002). Novel insights into the osmotic stress response of yeast. FEMS Yeast Res.

[B29] Ollivier H, Pichavant-Rafini K, Puill-Stephan E, Calves P, Nonnotte L, Nonnotte G (2006). Effects of hyposmotic stress on exocytosis in isolated turbot, Scophthalmus maximus, hepatocytes. J Comp Physiol [B].

[B30] Wu MH, Dimopoulos G, Mantalaris A, Varley J (2004). The effect of hyperosmotic pressure on antibody production and gene expression in the GS-NS0 cell line. Biotechnol Appl Biochem.

[B31] Jahic M, Veide A, Charoenrat T, Teeri T, Enfors SO (2006). Process technology for production and recovery of heterologous proteins with Pichia pastoris. Biotechnol Prog.

[B32] Fernandes AR, Mira NP, Vargas RC, Canelhas I, Sa-Correia I (2005). Saccharomyces cerevisiae adaptation to weak acids involves the transcription factor Haa1p and Haa1p-regulated genes. Biochem Biophys Res Commun.

[B33] Kapteyn JC, ter Riet B, Vink E, Blad S, De Nobel H, Van Den Ende H, Klis FM (2001). Low external pH induces HOG1-dependent changes in the organization of the Saccharomyces cerevisiae cell wall. Mol Microbiol.

[B34] Dorner A, Kaufman R (1994). The levels of endoplasmic reticulum proteins and ATP affect folding and secretion of selective proteins. Biologicals.

[B35] Nishikawa S, Fewell S, Kato Y, Brodsky J, Endo T (2001). Molecular chaperones in the yeast endoplasmic reticulum maintain the solubility of proteins for retrotranslocation and degradation. J Cell Biol.

[B36] Kozutsumi Y, Segal M, Normington K, Gething M, Sambrook J (1988). The presence of malfolded proteins in the endoplasmic reticulum signals the induction of glucose-regulated proteins. Nature.

[B37] Robinson A, Wittrup K (1995). Constitutive overexpression of secreted heterologous proteins decreases extractable BiP and protein disulfide isomerase levels in Saccharomyces cerevisiae. Biotechnol Prog.

[B38] Kauffman K, Pridgen E, Doyle Fr, Dhurjati P, Robinson A (2002). Decreased protein expression and intermittent recoveries in BiP levels result from cellular stress during heterologous protein expression in Saccharomyces cerevisiae. Biotechnol Prog.

[B39] Hohenblum H, Gasser B, Maurer M, Borth N, Mattanovich D (2004). Effects of gene dosage, promoters, and substrates on unfolded protein stress of recombinant Pichia pastoris. Biotechnol Bioeng.

[B40] Nyyssönen E, Keränen S (1995). Multiple roles of the cellulase CBHI in enhancing production of fusion antibodies by the filamentous fungus Trichoderma reesei. Curr Genet.

[B41] Pakula T, Salonen K, Uusitalo J, Penttilä M (2005). The effect of specific growth rate on protein synthesis and secretion in the filamentous fungus Trichoderma reesei. Microbiology.

[B42] Punt P, van Gemeren I, Drint-Kuijvenhoven J, Hessing J, van Muijlwijk-Harteveld G, Beijersbergen A, Verrips C, van den Hondel C (1998). Analysis of the role of the gene bipA, encoding the major endoplasmic reticulum chaperone protein in the secretion of homologous and heterologous proteins in black Aspergilli. Appl Microbiol Biotechnol.

[B43] Plemper RK, Bordallo J, Deak PM, Taxis C, Hitt R, Wolf DH (1999). Genetic interactions of Hrd3p and Der3p/Hrd1p with Sec61p suggest a retro-translocation complex mediating protein transport for ER degradation. J Cell Sci.

[B44] Sagt C, Müller W, van der Heide L, Boonstra J, Verkleij A, Verrips C (2002). Impaired cutinase secretion in Saccharomyces cerevisiae induces irregular endoplasmic reticulum (ER) membrane proliferation, oxidative stress, and ER-associated degradation. Appl Environ Microbiol.

[B45] Haynes CM, Titus EA, Cooper AA (2004). Degradation of misfolded proteins prevents ER-derived oxidative stress and cell death. Mol Cell.

[B46] Kincaid MM, Cooper AA (2007). ERADicate ER stress or die trying. Antioxid Redox Signal.

[B47] Ron D, Walter P (2007). Signal integration in the endoplasmic reticulum unfolded protein response. Nat Rev Mol Cell Biol.

[B48] Cox JS, Shamu CE, Walter P (1993). Transcriptional induction of genes encoding endoplasmic reticulum resident proteins requires a transmembrane protein kinase. Cell.

[B49] Cox JS, Walter P (1996). A novel mechanism for regulating activity of a transcription factor that controls the unfolded protein response. Cell.

[B50] Saloheimo M, Valkonen M, Penttila M (2003). Activation mechanisms of the HAC1-mediated unfolded protein response in filamentous fungi. Mol Microbiol.

[B51] Yoshida H, Matsui T, Yamamoto A, Okada T, Mori K (2001). XBP1 mRNA is induced by ATF6 and spliced by IRE1 in response to ER stress to produce a highly active transcription factor. Cell.

[B52] Sidrauski C, Cox JS, Walter P (1996). tRNA ligase is required for regulated mRNA splicing in the unfolded protein response. Cell.

[B53] Ruegsegger U, Leber JH, Walter P (2001). Block of HAC1 mRNA translation by long-range base pairing is released by cytoplasmic splicing upon induction of the unfolded protein response. Cell.

[B54] Mulder HJ, Saloheimo M, Penttila M, Madrid SM (2004). The transcription factor HACA mediates the unfolded protein response in Aspergillus niger, and up-regulates its own transcription. Mol Genet Genomics.

[B55] Arvas M, Pakula T, Lanthaler K, Saloheimo M, Valkonen M, Suortti T, Robson G, Penttila M (2006). Common features and interesting differences in transcriptional responses to secretion stress in the fungi Trichoderma reesei and Saccharomyces cerevisiae. BMC Genomics.

[B56] Guillemette T, van Peij N, Goosen T, Lanthaler K, Robson G, van den Hondel C, Stam H, Archer D (2007). Genomic analysis of the secretion stress response in the enzyme-producing cell factory Aspergillus niger. BMC Genomics.

[B57] Sims AH, Gent ME, Lanthaler K, Dunn-Coleman NS, Oliver SG, Robson GD (2005). Transcriptome analysis of recombinant protein secretion by Aspergillus nidulans and the unfolded-protein response in vivo. Appl Environ Microbiol.

[B58] Resina D, Bollók M, Khatri N, Valero F, Neubauer P, Ferrer P (2007). Transcriptional response of P. pastoris in fed-batch cultivations to Rhizopus oryzae lipase production reveals UPR induction. Microb Cell Fact.

[B59] Harding HP, Zhang Y, Ron D (1999). Protein translation and folding are coupled by an endoplasmic-reticulum-resident kinase. Nature.

[B60] Pakula TM, Laxell M, Huuskonen A, Uusitalo J, Saloheimo M, Penttila M (2003). The effects of drugs inhibiting protein secretion in the filamentous fungus Trichoderma reesei. Evidence for down-regulation of genes that encode secreted proteins in the stressed cells. J Biol Chem.

[B61] Al-Sheikh H, Watson AJ, Lacey GA, Punt PJ, MacKenzie DA, Jeenes DJ, Pakula T, Penttila M, Alcocer MJ, Archer DB (2004). Endoplasmic reticulum stress leads to the selective transcriptional downregulation of the glucoamylase gene in Aspergillus niger. Mol Microbiol.

[B62] Martinez IM, Chrispeels MJ (2003). Genomic analysis of the unfolded protein response in Arabidopsis shows its connection to important cellular processes. Plant Cell.

[B63] Robinson A, Hines V, Wittrup K (1994). Protein disulfide isomerase overexpression increases secretion of foreign proteins in Saccharomyces cerevisiae. Biotechnology (NY).

[B64] Harmsen M, Bruyne M, Raué H, Maat J (1996). Overexpression of binding protein and disruption of the PMR1 gene synergistically stimulate secretion of bovine prochymosin but not plant thaumatin in yeast. Appl Microbiol Biotechnol.

[B65] Kim M, Han K, Kang H, Rhee S, Seo J (2003). Coexpression of BiP increased antithrombotic hirudin production in recombinant Saccharomyces cerevisiae. J Biotechnol.

[B66] Lombraña M, Moralejo F, Pinto R, Martín J (2004). Modulation of Aspergillus awamori thaumatin secretion by modification of bipA gene expression. Appl Environ Microbiol.

[B67] Robinson A, Bockhaus J, Voegler A, Wittrup K (1996). Reduction of BiP levels decreases heterologous protein secretion in Saccharomyces cerevisiae. J Biol Chem.

[B68] van Gemeren I, Beijersbergen A, van den Hondel C, Verrips C (1998). Expression and secretion of defined cutinase variants by Aspergillus awamori. Appl Environ Microbiol.

[B69] van der Heide M, Hollenberg C, van der Klei I, Veenhuis M (2002). Overproduction of BiP negatively affects the secretion of Aspergillus niger glucose oxidase by the yeast Hansenula polymorpha. Appl Microbiol Biotechnol.

[B70] Smith J, Tang B, Robinson A (2004). Protein disulfide isomerase, but not binding protein, overexpression enhances secretion of a non-disulfide-bonded protein in yeast. Biotechnol Bioeng.

[B71] Conesa A, Jeenes D, Archer DB, van den Hondel CA, Punt PJ (2002). Calnexin overexpression increases manganese peroxidase production in Aspergillus niger. Appl Environ Microbiol.

[B72] Chung JY, Lim SW, Hong YJ, Hwang SO, Lee GM (2004). Effect of doxycycline-regulated calnexin and calreticulin expression on specific thrombopoietin productivity of recombinant Chinese hamster ovary cells. Biotechnol Bioeng.

[B73] Higgins MK, Demir M, Tate CG (2003). Calnexin co-expression and the use of weaker promoters increase the expression of correctly assembled Shaker potassium channel in insect cells. Biochim Biophys Acta.

[B74] Klabunde J, Kleebank S, Piontek M, Hollenberg CP, Hellwig S, Degelmann A (2007). Increase of calnexin gene dosage boosts the secretion of heterologous proteins by Hansenula polymorpha. FEMS Yeast Res.

[B75] Marechal A, Tanguay PL, Callejo M, Guerin R, Boileau G, Rokeach LA (2004). Cell viability and secretion of active proteins in Schizosaccharomyces pombe do not require the chaperone function of calnexin. Biochem J.

[B76] Parlati F, Dominguez M, Bergeron JJ, Thomas DY (1995). Saccharomyces cerevisiae CNE1 encodes an endoplasmic reticulum (ER) membrane protein with sequence similarity to calnexin and calreticulin and functions as a constituent of the ER quality control apparatus. J Biol Chem.

[B77] Arima H, Kinoshita T, Ibrahim HR, Azakami H, Kato A (1998). Enhanced secretion of hydrophobic peptide fused lysozyme by the introduction of N-glycosylation signal and the disruption of calnexin gene in Saccharomyces cerevisiae. FEBS Lett.

[B78] Song Y, Sata J, Saito A, Usui M, Azakami H, Kato A (2001). Effects of calnexin deletion in Saccharomyces cerevisiae on the secretion of glycosylated lysozymes. J Biochem (Tokyo).

[B79] Hayano T, Hirose M, Kikuchi M (1995). Protein disulfide isomerase mutant lacking its isomerase activity accelerates protein folding in the cell. FEBS Lett.

[B80] Bao W, Fukuhara H (2001). Secretion of human proteins from yeast: stimulation by duplication of polyubiquitin and protein disulfide isomerase genes in Kluyveromyces lactis. Gene.

[B81] Iwata T, Tanaka R, Suetsugu M, Ishibashi M, Tokunaga H, Kikuchi M, Tokunaga M (2004). Efficient secretion of human lysozyme from the yeast, Kluyveromyces lactis. Biotechnol Lett.

[B82] Vad R, Nafstad E, Dahl L, Gabrielsen O (2005). Engineering of a Pichia pastoris expression system for secretion of high amounts of intact human parathyroid hormone. J Biotechnol.

[B83] Gasser B, Maurer M, Gach J, Kunert R, Mattanovich D (2006). Engineering of Pichia pastoris for improved production of antibody fragments. Biotechnol Bioeng.

[B84] Inan M, Aryasomayajula D, Sinha J, Meagher M (2006). Enhancement of protein secretion in Pichia pastoris by overexpression of protein disulfide isomerase. Biotechnol Bioeng.

[B85] Shusta E, Raines R, Plückthun A, Wittrup K (1998). Increasing the secretory capacity of Saccharomyces cerevisiae for production of single-chain antibody fragments. Nat Biotechnol.

[B86] Damasceno L, Anderson K, Ritter G, Cregg J, Old L, Batt C (2006). Cooverexpression of chaperones for enhanced secretion of a single-chain antibody fragment in Pichia pastoris. Appl Microbiol Biotechnol.

[B87] Borth N, Mattanovich D, Kunert R, Katinger H (2005). Effect of increased expression of protein disulfide isomerase and heavy chain binding protein on antibody secretion in a recombinant CHO cell line. Biotechnol Prog.

[B88] Klabunde J, Hollenberg C, Gellissen G (2005). Impact of overexpressed secretory-pathway genes on the secretion of IFNgamma in Hansenula polymorpha applying an rDNA-cointegration approach for assessment. FEMS Yeast Res.

[B89] Derkx P, Madrid S (2001). The foldase CYPB is a component of the secretory pathway of Aspergillus niger and contains the endoplasmic reticulum retention signal HEEL. Mol Genet Genomics.

[B90] Wiebe M, Karandikar A, Robson G, Trinci A, Candia J, Trappe S, Wallis G, Rinas U, Derkx P, Madrid S (2001). Production of tissue plasminogen activator (t-PA) in Aspergillus niger. Biotechnol Bioeng.

[B91] Moralejo F, Watson A, Jeenes D, Archer D, Martín J (2001). A defined level of protein disulfide isomerase expression is required for optimal secretion of thaumatin by Aspegillus awamori. Mol Genet Genomics.

[B92] Gross E, Kastner D, Kaiser C, Fass D (2004). Structure of Ero1p, source of disulfide bonds for oxidative protein folding in the cell. Cell.

[B93] Lodi T, Neglia B, Donnini C (2005). Secretion of human serum albumin by Kluyveromyces lactis overexpressing KlPDI1 and KlERO1. Appl Environ Microbiol.

[B94] Travers K, Patil C, Wodicka L, Lockhart D, Weissman J, Walter P (2000). Functional and genomic analyses reveal an essential coordination between the unfolded protein response and ER-associated degradation. Cell.

[B95] Higashio H, Kohno K (2002). A genetic link between the unfolded protein response and vesicle formation from the endoplasmic reticulum. Biochem Biophys Res Commun.

[B96] Valkonen M, Penttilä M, Saloheimo M (2003). Effects of inactivation and constitutive expression of the unfolded- protein response pathway on protein production in the yeast Saccharomyces cerevisiae. Appl Environ Microbiol.

[B97] Valkonen M, Ward M, Wang H, Penttilä M, Saloheimo M (2003). Improvement of foreign-protein production in Aspergillus niger var. awamori by constitutive induction of the unfolded-protein response. Appl Environ Microbiol.

[B98] Mattanovich D, Borth N (2006). Applications of cell sorting in biotechnology. Microb Cell Fact.

[B99] Shusta EV, Kieke MC, Parke E, Kranz DM, Wittrup KD (1999). Yeast polypeptide fusion surface display levels predict thermal stability and soluble secretion efficiency. J Mol Biol.

[B100] Rakestraw JA, Baskaran AR, Wittrup KD (2006). A flow cytometric assay for screening improved heterologous protein secretion in yeast. Biotechnol Prog.

[B101] Wentz AE, Shusta EV (2007). A novel high-throughput screen reveals yeast genes that increase secretion of heterologous proteins. Appl Environ Microbiol.

[B102] Sauer M, Branduardi P, Gasser B, Valli M, Maurer M, Porro D, Mattanovich D (2004). Differential gene expression in recombinant Pichia pastoris analysed by heterologous DNA microarray hybridisation. Microb Cell Fact.

[B103] Gasser B, Sauer M, Maurer M, Stadlmayr G, Mattanovich D (2007). Transcriptomics-based identification of novel factors enhancing heterologous protein secretion in yeasts. Appl Environ Microbiol.

[B104] Hoffmann F, Rinas U (2004). Stress induced by recombinant protein production in Escherichia coli. Adv Biochem Eng Biotechnol.

[B105] Baneyx F, Mujacic M (2004). Recombinant protein folding and misfolding in Escherichia coli. Nat Biotechnol.

[B106] Sorensen HP, Mortensen KK (2005). Advanced genetic strategies for recombinant protein expression in Escherichia coli. J Biotechnol.

[B107] Sorensen HP, Mortensen KK (2005). Soluble expression of recombinant proteins in the cytoplasm of Escherichia coli. MicrobCell Fact.

[B108] Villaverde A, Carrio MM (2003). Protein aggregation in recombinant bacteria: biological role of inclusion bodies. Biotechnol Lett.

[B109] Carrio M, Gonzalez-Montalban N, Vera A, Villaverde A, Ventura S (2005). Amyloid-like properties of bacterial inclusion bodies. J Mol Biol.

[B110] Speed MA, Wang DI, King J (1996). Specific aggregation of partially folded polypeptide chains: the molecular basis of inclusion body composition. Nat Biotechnol.

[B111] Hart R, Rinas U, Bailey J (1990). Protein composition of Vitreoscilla hemoglobin inclusion bodies produced in Escherichia coli. J Biol Chem.

[B112] Neubauer A, Soini J, Bollok M, Zenker M, Sandqvist J, Myllyharju J, Neubauer P (2007). Fermentation process for tetrameric human collagen prolyl 4-hydroxylase in Escherichia coli: Improvement by gene optimisation of the PDI/beta subunit and repeated addition of the inducer anhydrotetracycline. J Biotechnol.

[B113] Rinas U, Bailey JE (1992). Protein compositional analysis of inclusion bodies produced in recombinant Escherichia coli. Appl Microbiol Biotechnol.

[B114] Rinas U, Boone TC, Bailey JE (1993). Characterization of inclusion bodies in recombinant Escherichia coli producing high levels of porcine somatotropin. J Biotechnol.

[B115] Rinas U, Bailey JE (1993). Overexpression of bacterial hemoglobin causes incorporation of pre-beta-lactamase into cytoplasmic inclusion bodies. Appl Environ Microbiol.

[B116] Rinas U, Hoffmann F, Betiku E, Estape D, Marten S (2007). Inclusion body anatomy and functioning of chaperone-mediated in vivo inclusion body disassembly during high-level recombinant protein production in Escherichia coli. J Biotechnol.

[B117] Allen SP, Polazzi JO, Gierse JK, Easton AM (1992). Two novel heat shock genes encoding proteins produced in response to heterologous protein expression in Escherichia coli. J Bacteriol.

[B118] Carrio MM, Villaverde A (2002). Construction and deconstruction of bacterial inclusion bodies. J Biotechnol.

[B119] Carrio MM, Villaverde A (2005). Localization of chaperones DnaK and GroEL in bacterial inclusion bodies. J Bacteriol.

[B120] Vallejo LF, Rinas U (2004). Strategies for the recovery of active proteins through refolding of bacterial inclusion body proteins. MicrobCell Fact.

[B121] Miot M, Betton JM (2004). Protein quality control in the bacterial periplasm. MicrobCell Fact.

[B122] Connolly L, De Las Penas A, Alba B, Gross C (1997). The response to extracytoplasmic stress in Escherichia coli is controlled by partially overlapping pathways. Genes Dev.

[B123] Hunke S, Betton JM (2003). Temperature effect on inclusion body formation and stress response in the periplasm of Escherichia coli. Mol Microbiol.

[B124] Zahrl D, Wagner M, Bischof K, Koraimann G (2006). Expression and assembly of a functional type IV secretion system elicit extracytoplasmic and cytoplasmic stress responses in Escherichia coli. J Bacteriol.

[B125] Duguay A, Silhavy T (2004). Quality control in the bacterial periplasm. Biochim Biophys Acta.

[B126] Jurgen B, Lin HY, Riemschneider S, Scharf C, Neubauer P, Schmid R, Hecker M, Schweder T (2000). Monitoring of genes that respond to overproduction of an insoluble recombinant protein in Escherichia coli glucose-limited fed-batch fermentations. Biotechnol Bioeng.

[B127] Lesley SA, Graziano J, Cho CY, Knuth MW, Klock HE (2002). Gene expression response to misfolded protein as a screen for soluble recombinant protein. Protein Eng.

[B128] Arsene F, Tomoyasu T, Bukau B (2000). The heat shock response of Escherichia coli. Int J Food Microbiol.

[B129] Bukau B (1993). Regulation of the Escherichia coli heat-shock response. Mol Microbiol.

[B130] Aris A, Corchero JL, Benito A, Carbonell X, Viaplana E, Villaverde A (1998). The expression of recombinant genes from bacteriophage lambda strong promoters triggers the SOS response in Escherichia coli. Biotechnol Bioeng.

[B131] Harcum SW, Bentley WE (1999). Heat-shock and stringent responses have overlapping protease activity in Escherichia coli. Implications for heterologous protein yield. Appl Biochem Biotechnol.

[B132] Schultz T, Martinez L, de MA (2006). The evaluation of the factors that cause aggregation during recombinant expression in E. coli is simplified by the employment of an aggregation-sensitive reporter. MicrobCell Fact.

[B133] Hoffmann F, Rinas U (2000). Kinetics of heat-shock response and inclusion body formation during temperature-induced production of basic fibroblast growth factor in high-cell-density cultures of recombinant Escherichia coli. Biotechnol Prog.

[B134] Strandberg L, Enfors SO (1991). Factors influencing inclusion body formation in the production of a fused protein in Escherichia coli. Appl Environ Microbiol.

[B135] Vasina JA, Baneyx F (1996). Recombinant protein expression at low temperatures under the transcriptional control of the major Escherichia coli cold shock promoter cspA. Appl Environ Microbiol.

[B136] Vasina JA, Baneyx F (1997). Expression of aggregation-prone recombinant proteins at low temperatures: a comparative study of the Escherichia coli cspA and tac promoter systems. Protein Expr Purif.

[B137] Vasina JA, Peterson MS, Baneyx F (1998). Scale-up and optimization of the low-temperature inducible cspA promoter system. Biotechnol Prog.

[B138] De Marco V, Stier G, Blandin S, de Marco A (2004). The solubility and stability of recombinant proteins are increased by their fusion to NusA. Biochem Biophys Res Commun.

[B139] Huang H, Liu J, de MA (2006). Induced fit of passenger proteins fused to Archaea maltose binding proteins. Biochem Biophys Res Commun.

[B140] Baneyx F, Palumbo JL (2003). Improving heterologous protein folding via molecular chaperone and foldase co-expression. Methods Mol Biol.

[B141] Hoffmann F, Rinas U (2004). Roles of heat-shock chaperones in the production of recombinant proteins in Escherichia coli. Adv Biochem Eng Biotechnol.

[B142] Schlieker C, Bukau B, Mogk A (2002). Prevention and reversion of protein aggregation by molecular chaperones in the E. coli cytosol: implications for their applicability in biotechnology. J Biotechnol.

[B143] Thomas JG, Ayling A, Baneyx F (1997). Molecular chaperones, folding catalysts, and the recovery of active recombinant proteins from E. coli. To fold or to refold. Appl Biochem Biotechnol.

[B144] de Marco A, de Marco V (2004). Bacteria co-transformed with recombinant proteins and chaperones cloned in independent plasmids are suitable for expression tuning. J Biotechnol.

[B145] de Marco A, Deuerling E, Mogk A, Tomoyasu T, Bukau B (2007). Chaperone-based procedure to increase yields of soluble recombinant proteins produced in E. coli. BMC Biotechnol.

[B146] de Marco A (2007). Protocol for preparing proteins with improved solubility by co-expressing with molecular chaperones in Escherichia coli. Nat Protoc.

[B147] Nishihara K, Kanemori M, Kitagawa M, Yanagi H, Yura T (1998). Chaperone coexpression plasmids: differential and synergistic roles of DnaK-DnaJ-GrpE and GroEL-GroES in assisting folding of an allergen of Japanese cedar pollen, Cryj2, in Escherichia coli. Appl Environ Microbiol.

[B148] Garcia-Fruitos E, Gonzalez-Montalban N, Morell M, Vera A, Ferraz RM, Aris A, Ventura S, Villaverde A (2005). Aggregation as bacterial inclusion bodies does not imply inactivation of enzymes and fluorescent proteins. MicrobCell Fact.

[B149] Garcia-Fruitos E, Carrio MM, Aris A, Villaverde A (2005). Folding of a misfolding-prone beta-galactosidase in absence of DnaK. Biotechnol Bioeng.

[B150] Gonzalez-Montalban N, Garcia-Fruitos E, Ventura S, Aris A, Villaverde A (2006). The chaperone DnaK controls the fractioning of functional protein between soluble and insoluble cell fractions in inclusion body-forming cells. MicrobCell Fact.

[B151] Martinez-Alonso M, Vera A, Villaverde A (2007). Role of the chaperone DnaK in protein solubility and conformational quality in inclusion body-forming Escherichia coli cells. FEMS MicrobiolLett.

[B152] Garcia-Fruitos E, Martinez-Alonso M, Gonzalez-Montalban N, Valli M, Mattanovich D, Villaverde A (2007). Divergent Genetic Control of Protein Solubility and Conformational Quality in Escherichia coli. J Mol Biol.

[B153] Carrio MM, Villaverde A (2003). Role of molecular chaperones in inclusion body formation. FEBS Lett.

[B154] Vera A, Aris A, Carrio M, Gonzalez-Montalban N, Villaverde A (2005). Lon and ClpP proteases participate in the physiological disintegration of bacterial inclusion bodies. J Biotechnol.

[B155] Mogk A, Schlieker C, Friedrich KL, Schonfeld HJ, Vierling E, Bukau B (2003). Refolding of substrates bound to small Hsps relies on a disaggregation reaction mediated most efficiently by ClpB/DnaK. J Biol Chem.

[B156] Mogk A, Deuerling E, Vorderwulbecke S, Vierling E, Bukau B (2003). Small heat shock proteins, ClpB and the DnaK system form a functional triade in reversing protein aggregation. Mol Microbiol.

[B157] Mogk A, Bukau B (2004). Molecular chaperones: structure of a protein disaggregase. Curr Biol.

[B158] Gonzalez-Montalban N, Garcia-Fruitos E, Villaverde A (2007). Recombinant protein solubility-does more mean better?. Nat Biotechnol.

[B159] Nakamoto H, Bardwell JC (2004). Catalysis of disulfide bond formation and isomerization in the Escherichia coli periplasm. Biochim Biophys Acta.

[B160] Hu X, O'Dwyer R, Wall JG (2005). Cloning, expression and characterisation of a single-chain Fv antibody fragment against domoic acid in Escherichia coli. J Biotechnol.

[B161] Maskos K, Huber-Wunderlich M, Glockshuber R (2003). DsbA and DsbC-catalyzed oxidative folding of proteins with complex disulfide bridge patterns in vitro and in vivo. J Mol Biol.

[B162] Qiu J, Swartz JR, Georgiou G (1998). Expression of active human tissue-type plasminogen activator in Escherichia coli. Appl Environ Microbiol.

[B163] Ostermeier M, De Sutter K, Georgiou G (1996). Eukaryotic protein disulfide isomerase complements Escherichia coli dsbA mutants and increases the yield of a heterologous secreted protein with disulfide bonds. J Biol Chem.

[B164] Zhan X, Schwaller M, Gilbert HF, Georgiou G (1999). Facilitating the formation of disulfide bonds in the Escherichia coli periplasm via coexpression of yeast protein disulfide isomerase. Biotechnol Prog.

[B165] Bessette PH, Aslund F, Beckwith J, Georgiou G (1999). Efficient folding of proteins with multiple disulfide bonds in the Escherichia coli cytoplasm. Proc Natl Acad Sci USA.

[B166] Stewart EJ, Aslund F, Beckwith J (1998). Disulfide bond formation in the Escherichia coli cytoplasm: an in vivo role reversal for the thioredoxins. EMBO J.

[B167] Jurado P, Ritz D, Beckwith J, de LV, Fernandez LA (2002). Production of functional single-chain Fv antibodies in the cytoplasm of Escherichia coli. J Mol Biol.

[B168] Santala V, Lamminmaki U (2004). Production of a biotinylated single-chain antibody fragment in the cytoplasm of Escherichia coli. J Immunol Methods.

[B169] Heo MA, Kim SH, Kim SY, Kim YJ, Chung J, Oh MK, Lee SG (2006). Functional expression of single-chain variable fragment antibody against c-Met in the cytoplasm of Escherichia coli. Protein Expr Purif.

[B170] Levy R, Weiss R, Chen G, Iverson BL, Georgiou G (2001). Production of correctly folded Fab antibody fragment in the cytoplasm of Escherichia coli trxB gor mutants via the coexpression of molecular chaperones. Protein Expr Purif.

[B171] Fernandez LA (2004). Prokaryotic expression of antibodies and affibodies. Curr Opin Biotechnol.

[B172] Terpe K (2006). Overview of bacterial expression systems for heterologous protein production: from molecular and biochemical fundamentals to commercial systems. Appl Microbiol Biotechnol.

[B173] van Wely K, Swaving J, Freudl R, Driessen A (2001). Translocation of proteins across the cell envelope of Gram-positive bacteria. FEMS Microbiol Rev.

[B174] Li W, Zhou X, Lu P (2004). Bottlenecks in the expression and secretion of heterologous proteins in Bacillus subtilis. Res Microbiol.

[B175] Bolhuis A, Tjalsma H, Smith HE, de Jong A, Meima R, Venema G, Bron S, van Dijl JM (1999). Evaluation of Bottlenecks in the Late Stages of Protein Secretion in Bacillus subtilis. Appl Environ Microbiol.

[B176] Yuan G, Wong S (1995). Isolation and characterization of Bacillus subtilis groE regulatory mutants: evidence for orf39 in the dnaK operon as a repressor gene in regulating the expression of both groE and dnaK. J Bacteriol.

[B177] Leskelä S, Wahlström E, Kontinen V, Sarvas M (1999). Lipid modification of prelipoproteins is dispensable for growth but essential for efficient protein secretion in Bacillus subtilis: characterization of the Lgt gene. Mol Microbiol.

[B178] Kontinen V, Sarvas M (1993). The PrsA lipoprotein is essential for protein secretion in Bacillus subtilis and sets a limit for high-level secretion. Mol Microbiol.

[B179] Hyyryläinen H, Bolhuis A, Darmon E, Muukkonen L, Koski P, Vitikainen M, Sarvas M, Prágai Z, Bron S, van Dijl J (2001). A novel two-component regulatory system in Bacillus subtilis for the survival of severe secretion stress. Mol Microbiol.

[B180] Westers H, Westers L, Darmon E, van Dijl J, Quax W, Zanen G (2006). The CssRS two-component regulatory system controls a general secretion stress response in Bacillus subtilis. FEBS J.

[B181] Duilio A, Tutino M, Marino G (2004). Recombinant protein production in Antarctic Gram-negative bacteria. Methods Mol Biol.

[B182] Parrilli E, Duilio A, Tutino ML, RMea (2008). Heterologous protein expression in psychrophilic hosts. Psychrophiles: from Biodiversity to Biotechnology.

[B183] Médigue C, Krin E, Pascal G, Barbe V, Bernsel A, Bertin P, Cheung F, Cruveiller S, D'Amico S, Duilio A (2005). Coping with cold: the genome of the versatile marine Antarctica bacterium Pseudoalteromonas haloplanktis TAC125. Genome Res.

[B184] Kiefhaber T, Rudolph R, Kohler H, Buchner J (1991). Protein aggregation in vitro and in vivo: a quantitative model of the kinetic competition between folding and aggregation. Biotechnology (NY).

[B185] de Pascale D, Cusano AM, Autore F, dP ParrilliE, Marino G, Tutino ML (2008). The cold-active Lip1 lipase from the Antarctic bacterium Pseudoalteromonas haloplanktis TAC125 is a member of a new bacterial lipolytic enzyme family. Extremophiles.

